# Four-dimensional flow CMR–based hemodynamic assessment of ALCAPA: A case report

**DOI:** 10.1016/j.radcr.2026.04.064

**Published:** 2026-05-15

**Authors:** Hideharu Oka, Kouichi Nakau, Yuki Shibagaki, Takafumi Nakata, Kenta Matsumoto, Sadahiro Nakagawa, Kunihiro Iwata, Satoru Takahashi

**Affiliations:** a Department of Pediatrics, Asahikawa Medical University, Hokkaido, Japan; b Section of Radiological Technology, Department of Medical Technology, Asahikawa Medical University Hospital, Hokkaido, Japan

**Keywords:** Anomalous left coronary artery from the pulmonary artery, Bland–White–Garland syndrome, Case report, Four-dimensional flow cardiac magnetic resonance, Maximum intensity projection images

## Abstract

In this report, we used 4-dimensional flow cardiac magnetic resonance (4D flow CMR) to quantify blood flow and assess the coronary artery course and ostium in Anomalous left coronary artery from the pulmonary artery (ALCAPA). In particular, maximum intensity projection images derived from 4D flow CMR clearly demonstrated dilation of the right coronary artery and the anomalous origin of the left coronary artery from the pulmonary artery. 4D flow CMR, which can be performed without contrast, may serve as a useful tool for understanding the pathology of and determining the treatment strategies for ALCAPA.

## Introduction

Anomalous left coronary artery from the pulmonary artery (ALCAPA), also referred to as Bland–White–Garland syndrome, is a rare congenital coronary artery anomaly that occurs in approximately 1 in 300,000 individuals [1]. Its presentation varies from asymptomatic incidental findings to cases presenting with chest symptoms. However, ALCAPA can cause myocardial infarction, left ventricular dysfunction, ventricular tachycardia, or even sudden cardiac death [1]. Thus, surgery is performed once the diagnosis is established [1,2]. Coronary computed tomography angiography is considered as the most suitable modality for visualizing the entire course of coronary arteries [2]. However, it cannot measure the direction of coronary blood flow or the pulmonary-to-systemic blood flow ratio (Qp/Qs), making it difficult to assess the hemodynamics of ALCAPA. Conversely, coronary angiography via cardiac catheterization is commonly used to assess the coronary artery course and Qp/Qs. Nevertheless, its invasive nature has led to a decline in its application [2]. Although magnetic resonance imaging (MRI) allows for the functional assessment of the heart and evaluation for myocardial ischemia, it is not commonly used [2,3]. This is because it requires a long examination time for cross-sectional settings to evaluate blood flow and comprehensively visualizing the entire course of the coronary arteries is difficult. Recently, 4-dimensional flow cardiac magnetic resonance (4D flow CMR) has been developed to evaluate the direction and volume of coronary blood flow with simple conditions. Herein, we present a case of ALCAPA in which we were able to visualize the course and ostium of the coronary arteries from maximum intensity projection (MIP) images by performing quantitative blood flow assessment using 4D flow CMR.

## Case presentation

**Fig. 1:**
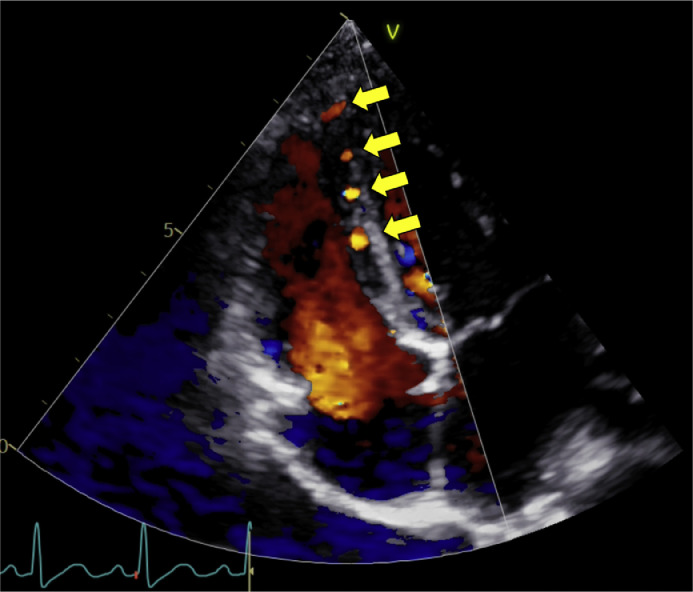
Echocardiography showed the characteristic Christmas tree pattern of anomalous left coronary artery from the pulmonary artery (yellow arrows).

**Fig. 2:**
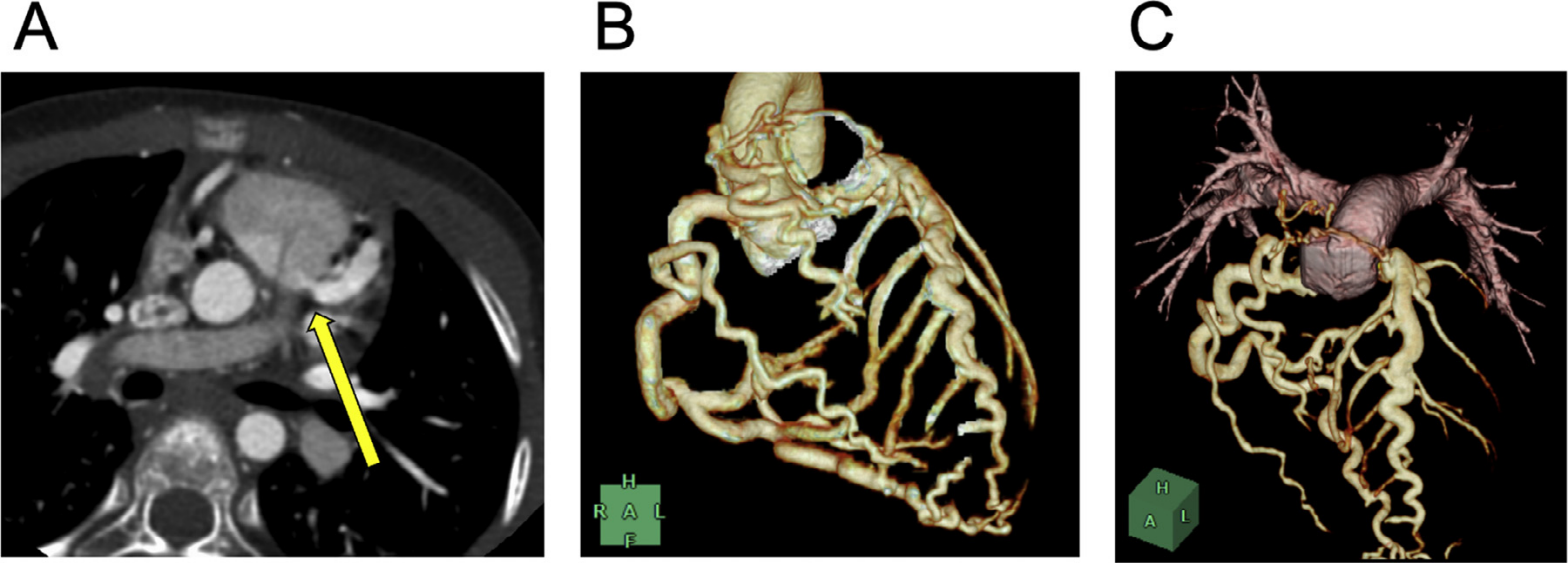
(A) Axial computed tomography showed the left coronary artery origin opened into the pulmonary artery (yellow arrows). (B-C) Multiplanar virtual reconstruction images showed dilatation and tortuosity of the RCA, showing communication between the RCA and the left anterior descending artery via the septal branch, and a collateral circulation connecting the RCA origin to the left main coronary trunk. RCA, right coronary artery.

**Fig. 3:**
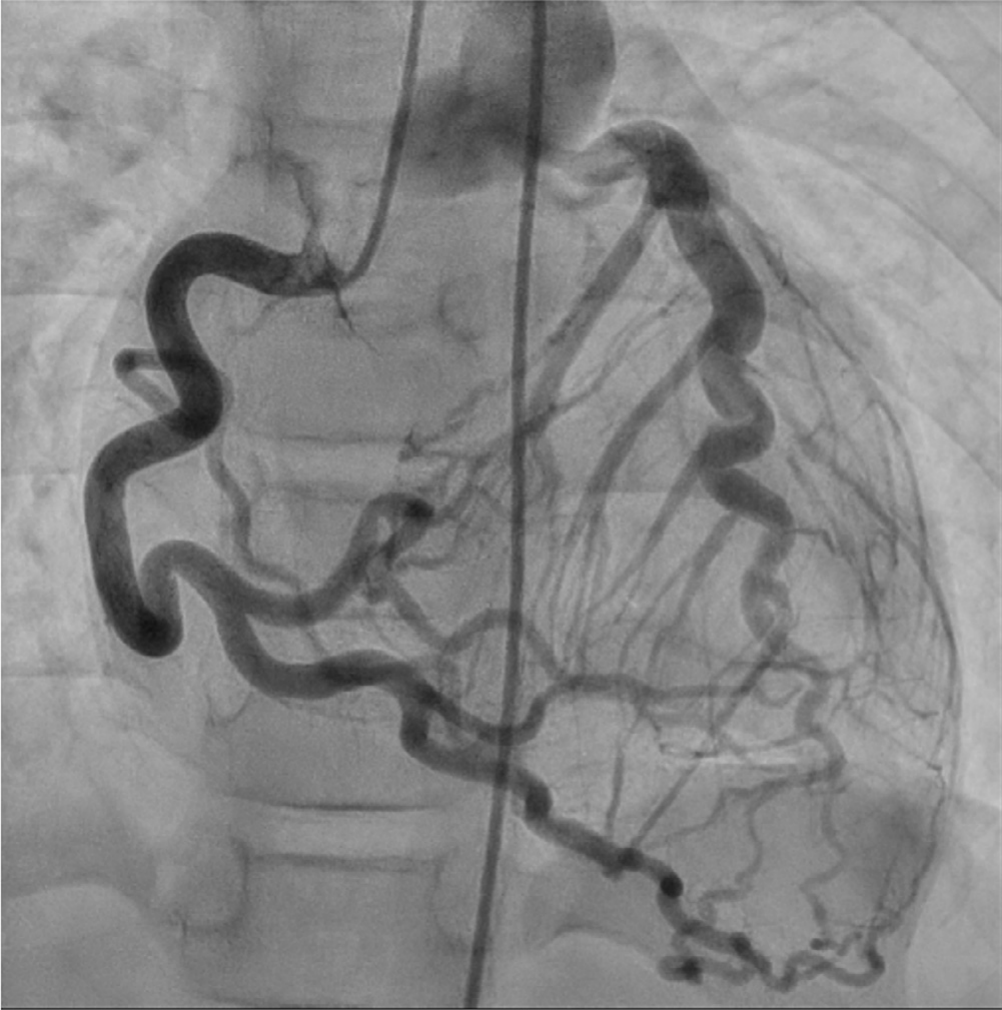
Angiography of the right coronary artery. The right coronary artery blood flow to the left coronary artery via the septal branch and collateral circulation. The left coronary artery showed both antegrade flow and flow being stolen into the pulmonary artery.

**Fig. 4:**
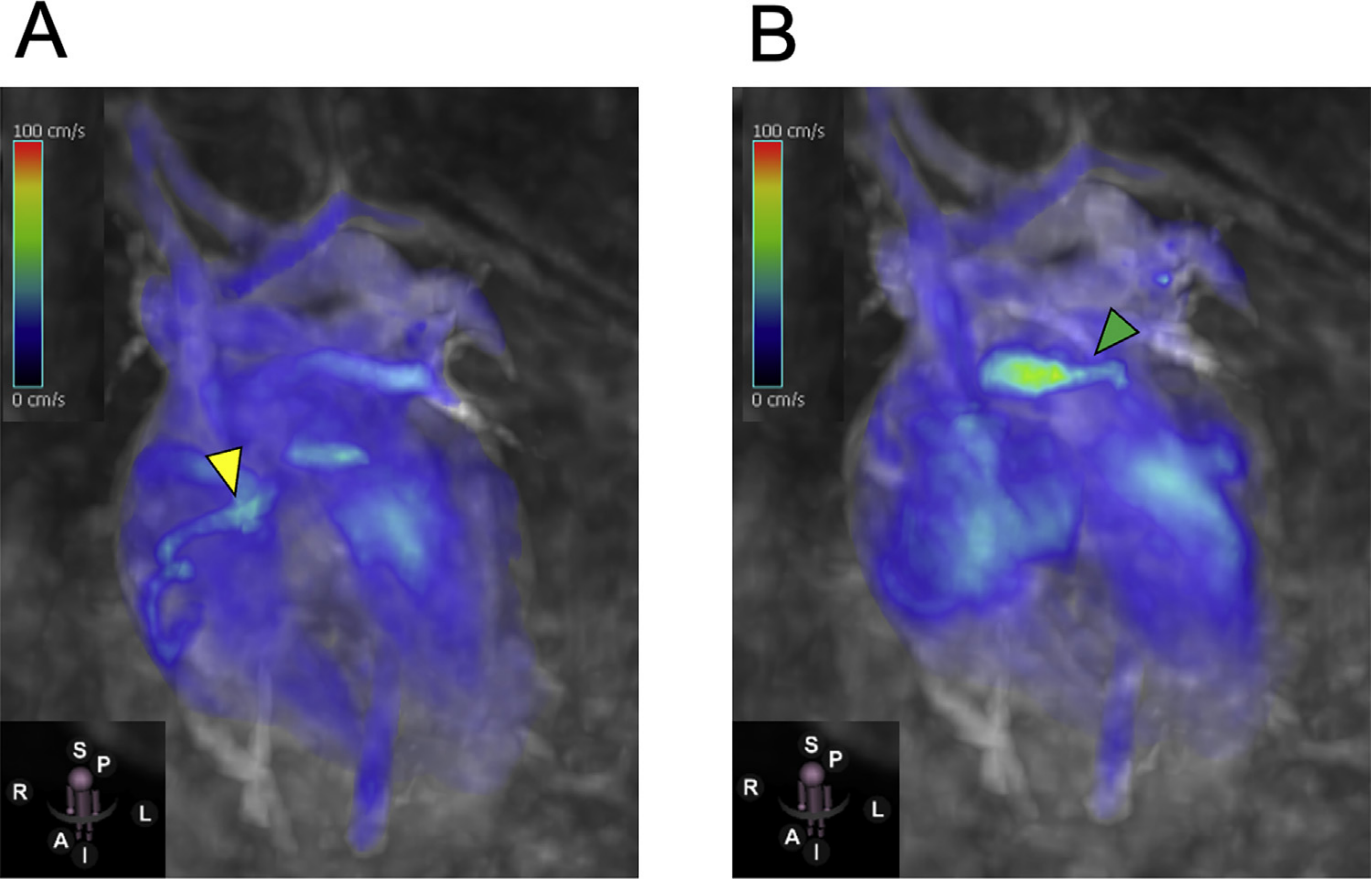
Four-dimensional flow cardiac magnetic resonance vector mapping. (A) The yellow arrowhead marked right coronary flow, and (B) the green arrowhead marked left coronary flow. Blood flow from the left coronary artery entered the pulmonary artery.

**Fig. 5:**
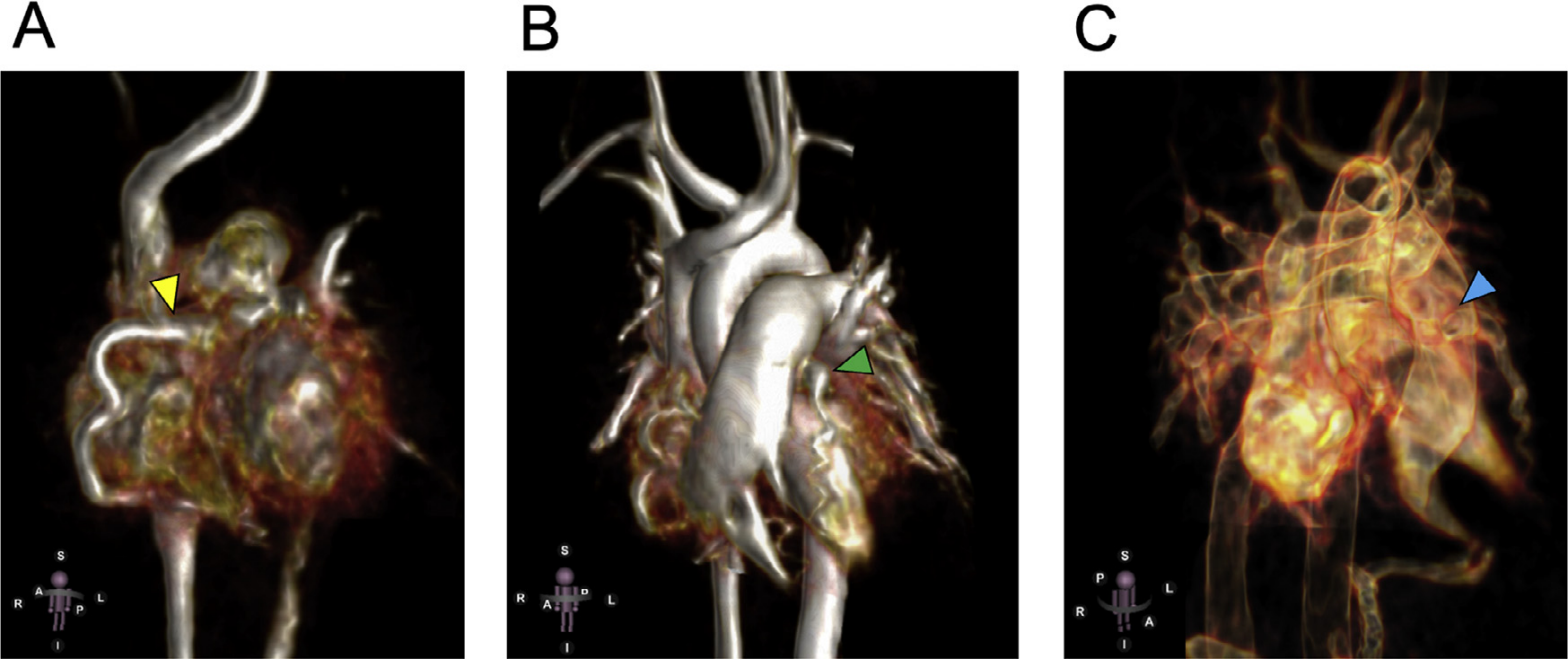
Four-dimensional flow cardiac magnetic resonance maximum intensity projection images. (A) The yellow arrowhead marked right coronary flow, and (B) the green arrowhead marked left coronary flow. (C) Observation in vascular mode showed that the left coronary artery originates from the pulmonary artery (the blue arrowhead).

The patient was a 6-year-old girl (height, 115 cm; weight, 19.6 kg). Electrocardiography screening during her first grade at elementary school led to a visit to a previous physician because of suspected left ventricular hypertrophy. Echocardiogram revealed an abnormal coronary artery course, prompting referral to our hospital. No abnormalities were observed during the perinatal period, and the patient had never experienced chest symptoms, fatigue, or reduced exercise tolerance. Electrocardiogram showed sinus rhythm at a heart rate of 127 bpm, with no findings suggestive of left ventricular hypertrophy and no Q waves or ST segment depression. Meanwhile, echocardiography revealed an enlargement of the right coronary artery (RCA; 4.7 mm, z-score 6.61). Moreover, the origin of the left coronary artery (LCA) could not be identified. Shunt flow was present into the pulmonary artery. The 4-chamber view showed the characteristic Christmas tree pattern of ALCAPA (Fig. 1). No mitral regurgitation was present, and there were no findings of left ventricular enlargement [left ventricular diastolic diameter: 38.2 mm (103% of the normal value)]. Left ventricular ejection fraction was 65%, indicating good cardiac function. However, coronary computed tomography angiography revealed dilatation and tortuosity of the RCA, showing communication between the RCA and the left anterior descending artery via the septal branch, and a collateral circulation connecting the RCA origin to the left main coronary trunk. The LCA origin opened into the pulmonary artery (Figs. 2A–C). Cardiac catheterization revealed right coronary angiography demonstrating blood flow to the LCA via the septal branch and collateral circulation (Fig. 3). The LCA showed both antegrade flow and flow being stolen into the pulmonary artery. 4D flow CMR was acquired and analyzed using previously published parameters [4]. The patient’s right ventricular and left ventricular cardiac output were 30.9 mL per heartbeat (3.7 L/min) and 36.6 mL per heartbeat (4.4 L/min), respectively. RCA flow was 4.9 mL per heartbeat (0.6 L/min). Meanwhile, LCA flow originating from the pulmonary artery was retrograde at 4.1 mL per heartbeat (0.5 L/min). Qp was defined as the sum of right ventricular output and retrograde LCA flow, and Qs as systemic flow with RCA flow subtracted, yielding a Qp/Qs ratio of 1.1. (Fig. 4A, B, Supplementary Video 1). Subsequently, MIP images confirmed enlargement of the RCA and LCA originating from the pulmonary artery (Fig. 5A, B). Image processing enabled visualization of the intravascular lumen, providing a clear depiction of the ostium of the LCA (Fig. 5C, Supplementary Video 2). The surgical plan has been established, and the patient is currently awaiting surgery.

## Discussion

We were able to quantitatively assess the hemodynamics of ALCAPA and evaluate the coronary artery course and ostium using 4D flow CMR. Because ALCAPA is a rare condition, few reports have utilized 4D flow CMR to evaluate this condition. Only case reports assessing quantitative blood flow or retrograde flow in the LCA have been published [5,6]. Moreover, we did not find reports describing the coronary artery course or LCA origin as we did by 4D flow CMR. The quantitative assessment of blood flow is important because ALCAPA can cause heart failure [2,3]. The ability to perform non-contrast examination avoids post-examination fluid volume increase because of contrast agents, thereby reducing the burden on the heart. Furthermore, myocardial fibrosis reportedly develops in ALCAPA because of myocardial ischemia caused by coronary steal [3]. Considering that the relationship between myocardial fibrosis and coronary steal fraction remains unclear, performing blood flow assessment using 4D flow CMR may enable the prediction of future myocardial fibrosis, offering promise for future research. Furthermore, capturing the appearance of the LCA origin in ALCAPA is crucial for establishing surgical treatment plans [7]. In particular, MIP 3-dimensional images clearly delineate the spatial relationships among the ALCAPA, aorta, and pulmonary artery. Moreover, because intramural coronary arteries can rarely occur in ALCAPA [7], these images may also be useful for their identification. The ability to identify the course of abnormal vessels using MIP images from 4D flow CMR is beneficial. As a non-contrast imaging technique, 4D flow CMR can be applied to patients with impaired renal function. Furthermore, it can capture the vascular course even in relatively small children, including our patient. Although MRI is not commonly used as a diagnostic tool for ALCAPA, we believe that its adoption for future examinations is promising because of the advantages outlined above.

4D flow CMR can be easily performed once certain configuration parameters are established. Furthermore, it is possible to identify the entire course and origin of coronary arteries by applying MIP processing. Moreover, these analyses often do not require excessive time. However, a significant issue of 4D flow CMR is its long acquisition time. Regarding this point, recent reports describe the application of compressed sensing techniques to 4D flow CMR, achieving approximately a 70% reduction in acquisition time [8]. If similar techniques become widely adopted, further improvements in examination time can be anticipated. Furthermore, 4D flow CMR has several limitations, including potential underestimation of flow in tortuous vessels and the need for sedation in young children. Accurate assessment of blood flow in small vessels remains challenging, and results should be interpreted in conjunction with clinical data. Shortening acquisition time using compressed sensing techniques may help reduce sedation requirements. Improving image quality and reducing scan time remain key challenges for 4D flow CMR.

## Conclusion

4D flow CMR enabled the quantitative assessment of blood flow, confirmation of coronary artery course, and identification of the LCA ostium in ALCAPA. This technique can be used as a diagnostic tool for understanding the pathology of and determining treatment strategies for ALCAPA.

## Ethics approval

This study was conducted in compliance with the standards of the Declaration of Helsinki and the current ethical guidelines and was approved by our institutional ethics board (Approval Number 19250).

## Patient consent

Written informed consent was obtained from the parents for publication of this case report in line with COPE guidance.

## Appendix Supplementary materials

Video 1. Four-dimensional flow cardiac magnetic resonance vector mapping movie. Image, video 1 dummy alt text

Video 2. Four-dimensional flow cardiac magnetic resonance maximum intensity projection movie. Image, video 2 dummy alt text
